# Patency of Posterior Circulation Branches Covered by Flow Diverter Device: A Hemodynamic Study

**DOI:** 10.3389/fneur.2019.00658

**Published:** 2019-06-19

**Authors:** Xinzhi Wu, Zhongbin Tian, Jian Liu, Yisen Zhang, Wenqiang Li, Ying Zhang, Junfan Chen, Yangyang Zhou, Xinjian Yang, Shiqing Mu

**Affiliations:** Department of Interventional Neuroradiology, Beijing Neurosurgical Institute, Beijing Tiantan Hospital, Capital Medical University, Beijing, China

**Keywords:** flow diverter device, pipeline, posterior circulation, side branch, hemodynamic

## Abstract

**Objective:** Flow diverter devices are increasingly used in the treatment of posterior circulation aneurysms, sometimes necessarily involving ostia of side branches and perforators. The aim of this study was to identify the hemodynamic influence of flow diverters on side branches and perforators of the posterior circulation.

**Methods:** We performed a retrospective study of consecutive patients treated by a flow diverter device for posterior circulation aneurysms with anterior inferior cerebellar artery (AICA) or posterior inferior cerebellar artery (PICA) involvement. Computational fluid dynamics (CFD) were used to discern hemodynamic changes of branches after deployment of the flow diverter.

**Results:** We studied 18 branches from 17 patients (mean age, 50.72 ± 8.17 years). No branches were occluded on immediate angiography and later follow-up. Average flow velocity in aneurysms decreased from 0.077 ± 0.065 m/s to 0.025 ± 0.025 m/s (*p* < 0.01). Average flow velocity in branch ostia decreased from 0.29 ± 0.14 m/s to 0.27 ± 0.16 m/s (*p* = 0.189). The difference in flow velocity reduction ratio between aneurysms and branches was statistically significant (68.8 vs. 9.5%; *p* < 0.001). The mean pressure in branch ostia increased from 10,717.4 ± 489.0 to 10,859.0 ± 643.4 Pa (*p* < 0.01).

**Conclusion:** While a flow diverter device is capable of slowing down aneurysmal inflow, it is unable to block the flow into branches and perforators when used in the treatment of posterior circulation aneurysms; flow velocity in branches even increased in some cases. With a low branch occlusion ratio, it may be acceptable to cover posterior circulation branches and perforators if unavoidable.

## Introduction

Flow diverter devices (FDs) are commonly used for the treatment of intracranial aneurysms (1, 2). Aneurysmal inflow can be altered by an FD and the process of thrombogenesis promoted. Aneurysms treated by FDs are more inclined to achieve complete occlusion compared with other endovascular therapies. The 5-year occlusion rate was recently reported as 95.2% (3). The pipeline embolization device (PED; Medtronic, Minneapolis, MN, USA) is a type of FD initially approved to treat internal carotid artery aneurysms, for which it was demonstrated to be safe and effective (4, 5). Since then, the PED has also been increasingly used for posterior circulation aneurysms. However, the safety of its application in these cases has not been well-defined, and there are reports of deaths and brainstem infarcts (6, 7).

A previous study revealed that deployment of the PED in posterior circulation leads to higher risk of ischemic complications in comparison with anterior circulation (1). After deployment of the PED, the origin of anterior inferior cerebellar artery (AICA), posterior inferior cerebellar artery (PICA), and basilar perforating arteries sometimes became involved. Considering that the AICA and PICA supply the cerebellum and rich perforating vessels that supply vital brainstem structures, coverage of branches was considered culpable for the infarcts and deaths after PED deployment. For this reason, the possibility of the PED blocking blood flow into branches demands a timely solution.

As a mature technology, computational fluid dynamics (CFD) is widely used to illustrate the blood flow status of intracranial arteries (8). The aim of this study was to ascertain the hemodynamic changes in covered branches and perforators by means of CFD simulations after deployment of the PED.

## Materials and Methods

### Study Population

From January 2016 to December 2018, 18 branches of 17 patients were collected retrospectively. In the case of perforators not being visible in three-dimensional (3D) reconstruction geometry, we accepted AICAs and PICAs as representing all branches and perforators. The inclusion criteria were: (1) unruptured posterior circulation aneurysms; (2) the aneurysms were treated by only one PED, and AICA or PICA were covered by the stent at the same time; (3) a reconstructed 3D model of the ipsilateral vertebral artery was acquired; (4) the landing zone of the PED had to be identified on digital subtraction angiography (DSA) images to ensure that branches were covered by the PED; (5) patients were followed up with a control angiogram. The exclusion criteria were (1) ruptured aneurysms and (2) traumatic aneurysms. All patients received aspirin (100 mg) and clopidogrel (75 mg) for 5 days before treatment. Platelet function assessment was performed by thromboelastography (TEG) before treatment, and the target platelet inhibition was between 30 and 90%. If inhibition was <30%, clopidogrel was replaced by prasugrel, and if inhibition higher than 90%, only clopidogrel was maintained until inhibition fell below 90%. During the procedure, the patients received a maintained intravenous heparin (70 U/kg). After treatment, the dual antiplatelet therapy was maintained for 6 months and aspirin indefinitely. The study was approved by the ethics board of our hospital, and all patients agreed to take part and gave written informed consent.

### Geometry and Hemodynamic Modeling

First, 3D aneurysm geometries were reconstructed from DSA images. Second, the geometries were repaired, cut, and smoothed using Geomagic Studio (version 12.0; Geomagic, Research Triangle Park, NC, USA), after which the surface geometries were saved as standard tessellation language (STL) format files. Third, the process of pipeline stent implantation was simulated using an in-house virtual stent-deployment technique described previously (9). There were 3 major steps. (1) In the pre-processing stage, vessel-specific initialization was used to isolate the parent vessel and a simplex mesh was created to fit the vessel according to its centerline. The maximum inscribed sphere diameter was then extracted inside the parent vessel along its centerline using MATLAB (R2013a; The Mathworks, Natick, MA, USA) and placed in a series of circles. (2) The simplex mesh inside the parent vessel was expanded to enable the deployed simplex mesh to closely adjoin the wall of the parent vessel. (3) In the post-processing stage, the pipeline stent vertex coordinates were determined on the deployed simplex mesh according to the stent pattern. Using Abaqus/explicit 6.12 software (Simulia, Providence, RI, USA), the vertex coordinates were then connected to form distinct wire curves. Thereafter, using the CAD program Creo Parametric 2.0 (PTC, Needham, MA, USA), these wire curves were swept into 3D structures to create a 3D solid stent. This 3D solid stent was placed into the original 3D vessel geometry and meshed together with it.

The coiled aneurysm sac was simulated according to a porous medium model described previously (10). We used the flowing algebraic equation: volume of the coil = π × (diameter of coil/2)^2^ × (length of the coil). We defined the ratio of the volume of the coils to volume of the aneurysms as the packing density.

The process of CFD modeling was described in our previous studies (11, 12). The deployed stent and 3D vessel geometry were imported into the automatic mesh generation software ICEM CFD version 14.0 (ANSYS, Canonsburg, PA, USA) to create finite-volume element grids for CFD simulation. The maximum element size was set at 0.2 mm. To present the geometry of the pipeline stent sufficiently, we set the element size on the stent at 0.01 mm (approximately one-third the width of the strut of the pipeline stent) (13). The models of untreated cases consisted of 1 million tetrahedral elements, and the model of treated cases consisted of 5 million tetrahedral elements. The ANSYS CFX 14.0 was then used to solve the flow-governing Navier-Stokes equations. The walls of blood vessels were assumed to be rigid, with no-slip boundary conditions. The blood flow was assumed to be laminar, homogeneous, incompressible Newtonian fluid. The dynamic viscosity and density of the blood flow was set at 0.004 kg/m/s and 1,060 kg/m^3^, respectively. Using transcranial Doppler imaging, we obtained a representative pulsatile period velocity profile from a normal human, which was set as the inflow boundary condition. The pressure distribution along the parent artery, branch vessels, and in the aneurysm was then computed using the drop in pressure calculated during the CFD simulations with respect to *p* = 10,000 Pa prescribed at the outlet (14, 15). Flow waveforms were scaled to achieve a mean inlet wall shear stress of 1.5 Pa under pulsatile conditions (15). Traction-free boundary conditions were implemented at the outlet. To avoid initial transients, two complete cardiac cycles were computed and data from the second cycle were gathered.

### Flow Velocity and Pressure Measurement

The flow velocity and pressure of the branch before and after treatment were measured at a transection of the branch's ostium at peak systole. Enlargement of the branch's origin was avoided when obtaining the transection. The flow velocity of aneurysms was defined as the average velocity of the entire aneurysm at peak systole.

### Statistical Analysis

Statistical analysis was performed using SPSS Statistics for Windows, version 17.0 (SPSS, Chicago, IL, USA). All quantitative hemodynamic parameters (before and after stent implantation) were summarized as the mean ± SD if normally distributed, and analyzed with the paired-samples *t* test. Statistical significance was considered at a *p* value of < 0.05.

## Results

### Basic Information

There are 18 branches from 17 patients recruited for this study. A summary of demographic data for the 17 patients included in this study is provided in Table 1. All branches remained patent at follow-up, with 15 (83.3%) aneurysms completely occluded and 3 partially occluded. No patients developed new neurological defects associated with branch occlusion.

**Table 1:** Demographics and clinical characteristics.

**Characteristic**	**Value**
No. of patients	17
No. of branches covered	18
Mean age ± standard deviation (years)	50.7 ± 8.2
**SEX**	
Male	10
Female	7
Mean aneurysm size (mm)	8.3 ± 4.8
**COVERED BRANCHES**	
AICA	2 (11.1%)
PICA	16 (88.9%)
**TREATMENT STRATEGY**	
PED	14 (82.4%)
PED + coil	3 (17.6%)
Mean follow-up time (months)	6.3 ± 1.8

### Flow Patterns

**Figure 1:**
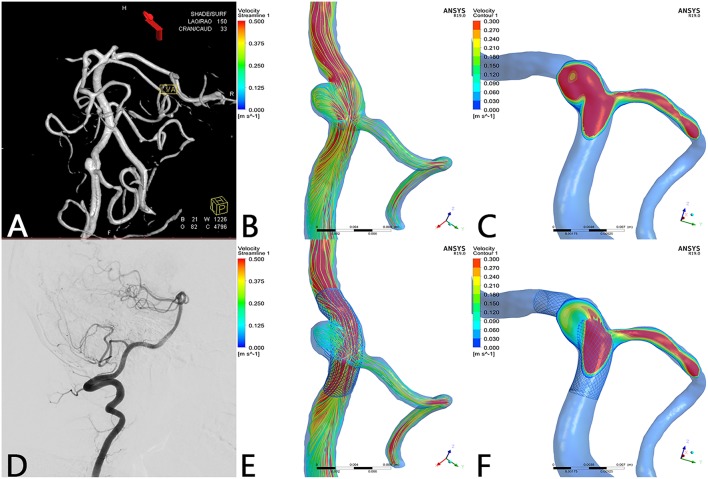
Case of a left vertebral artery aneurysm treated by a PED. **(A)** Left vertebral artery angiogram showing a V3 segment aneurysm. **(B)** Preoperative streamline of aneurysm and PICA. **(C)** Preoperative velocity contour of aneurysm and PICA. **(D)** Angiogram at 7-month follow-up showed that the aneurysm reached complete occlusion with patency of PICA. **(E)** Postoperative streamline of aneurysm and PICA. The streamline in the aneurysm became sparser compared with preoperatively. However, the streamline in PICA remained nearly unchanged compared with **(B)**. **(F)** Post-operative velocity contour of aneurysm and PICA. Compared with **(C)**, flow velocity in the aneurysm slowed down markedly, whereas flow velocity in the PICA merely decreased slightly at ostium.

**Figure 2:**
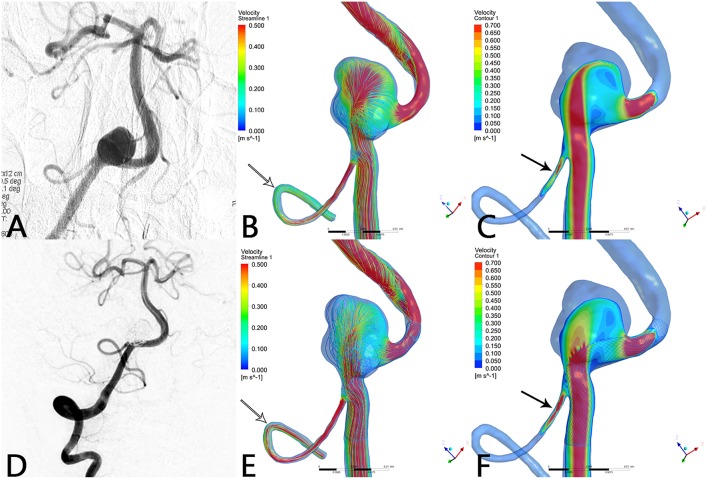
Case of a right vertebral artery aneurysm treated by PED. **(A)** Right vertebral artery angiogram showing a V4 segment aneurysm. **(B)** Preoperative streamline of aneurysm and PICA. **(C)** Preoperative velocity contour of aneurysm and PICA. **(D)** Angiogram at 6-month follow-up shows that the aneurysm reached complete occlusion with patency of PICA. **(E)** Postoperative streamline of aneurysm and PICA. The streamline in the aneurysm became sparser compared with preoperatively. Unexpectedly, the streamline in PICA increased (white arrow) compared with **(B)**. **(F)** Post-operative velocity contour of aneurysm and PICA. Compared with **(C)**, flow velocity in the aneurysm slowed down markedly, whereas flow velocity in PICA increased substantially (black arrow).

Before treatment, the streamlines in the geometry filled the parent artery, aneurysm (Figure 1A), and covered branches (Figure 1B). After the PED was deployed, the streamline into the aneurysm was reduced and flow velocity in the aneurysm decreased (Figure 1E). The decline in velocity can be visualized in the contrast between Figures 1C,F. Simultaneously, flow velocity in covered branches decreased slightly (Figures 1C,F). In some cases, however, the flow velocity did not decline and an increase in flow velocity was apparent (Figures 2C,F).

### Hemodynamic Changes

**Figure 3:**
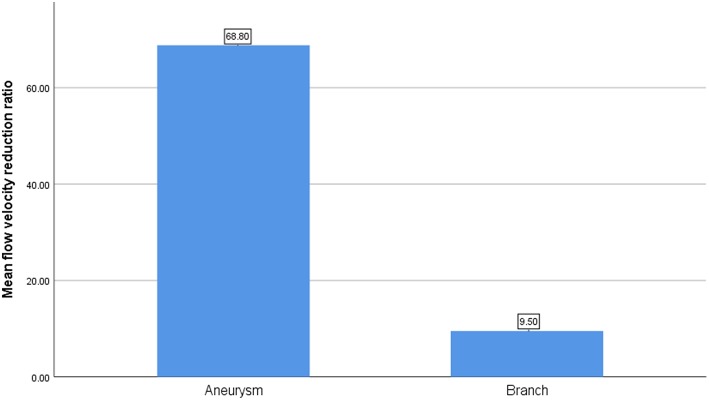
The comparison of mean flow velocity reduction ratio between aneurysm and branch.

**Figure 4:**
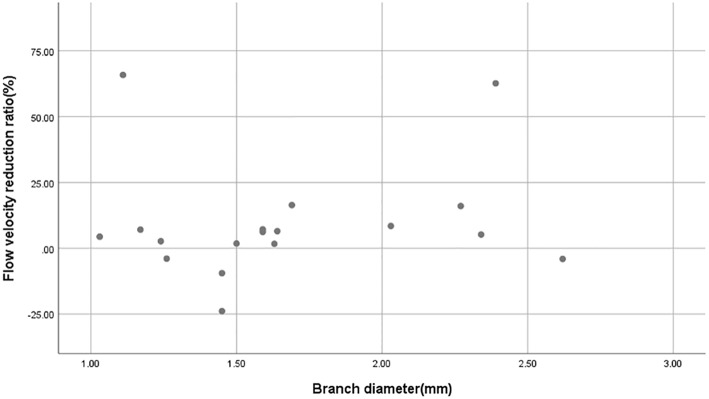
Scatterplot of blood flow reduction ratio distributions derived from different branch diameters. The scatterplot shows that there is no linear relationship between branch diameter and blood flow reduction ratio.

After the CFD simulation, we calculated the average blood flow velocity of branch ostium at peak systole. Before the operation this was 0.29 ± 0.14 m/s and postoperatively, 0.27 ± 0.16 m/s (*p* = 0.189). Before and after stent implantation, the average velocity of aneurysm at peak systole was 0.077 ± 0.065 m/s and 0.025 ± 0.025 m/s (*p* < 0.01), respectively. The mean flow velocity reduction ratio in aneurysms is apparently higher than that in branches (Figure 3). The mean pressure in branch ostia at peak systole increased from 10,717.4 ± 489.0 to 10,859.0 ± 643.4 Pa (*p* < 0.01). There was no linear relationship between blood flow reduction ratios and side branch diameters (Figure 4).

## Discussion

Aneurysms of the posterior circulation are challenging to treat via either microsurgical clipping or endovascularly. With several studies having revealed that microsurgical treatment has a worse outcome than an endovascular approach (16, 17), endovascular treatment is the preferred option by neurosurgeons. The traditional endovascular treatment strategy consists of coiling, stent-assisted coiling, and parent vessel occlusion, but currently FD is emerging as a new method of endovascular treatment. The PED diverts blood flow away from the aneurysm, supports neointimal overgrowth, accelerates thrombus formation, and finally excludes the aneurysm from the parent artery. Considering these effects of PED on the aneurysm, whether branches covered by the PED will become occluded and the mechanism of branch occlusion remains an open question.

In this series of 18 branches covered by a PED, flow diversion of posterior circulation aneurysms showed a satisfactory occlusion rate with no covered branches being occluded. Our CFD simulation results showed that the blood flow velocity in aneurysms changed markedly (from 0.077 ± 0.065 m/s to 0.025 ± 0.025 m/s; *p* < 0.01) after PED deployment. By contrast (Figure 3), the blood flow velocity in covered branches decreased from 0.29 ± 0.14 m/s to 0.27 ± 0.16 m/s (*p* = 0.189), but without statistical significance. The flow velocity reduction ratio [(pre-treatment parameter—post-treatment parameter)/pretreatment parameter ×100%] in the covered branches was 9.5 ± 21.3%, while the flow velocity reduction ratio in the aneurysm was 68.8 ± 13.4%. The traditional view is that a larger branch diameter will lead to a higher flow velocity reduction ratio, but according to the scatterplot in Figure 4 there is no linear relationship between them. These CFD results are consistent with follow-up angiography. Flow velocity in the aneurysm was reduced by up to 68.8 ± 13.4%, an alteration promoting complete occlusion of aneurysms (Figures 1D, 2D). Meanwhile, covered branches had relatively lower reduction in blood flow velocity and remained patent at follow-up (Figures 1D, 2D). In addition, we found that the pressure in branch ostia increased from 10,717.4 ± 489.0 Pa to 10,859.0 ± 643.4 Pa (*p* < 0.01), which offers a reasonable explanation for the patency of covered branches. The increased flow velocity of the PICA shown in Figure 2 is further evidence of the patency of covered branches. We consider that under the influence of the pipeline stent and coils, the flow into the aneurysm was partly diverted to the PICA, thus increasing flow velocity. Our CFD simulation results and follow-up angiography indicate that the FD has a comparatively low blocking effect on the blood flow of covered branches, and the reduced flow velocity in branches can be neutralized by the slightly increased pressure.

There is similar research in the literature. Hu et al. (18) concluded that reduced blood flow may not be the dominant factor that leads to side branch-related infarction. They simulated the deployment of the PED and covered 31 small branches using CFD by calculating the hemodynamic parameters before and after deployment of the pipeline stent, whereby the flow reduction ratio in AICAs was 3.62 ± 1.94%. The weakness of this study was that they did not combine their CFD results with clinical data. Mazur et al. (19) reported 11 cases of vertebral artery aneurysms treated by the pipeline embolization device. The device covered the origin of the PICA in each case, and no covered branch occlusion was identified at follow-up angiography.

Dai et al. (20) implanted single-, double-, and triple-telescoped/overlapped FDs in 22 rabbit aortas to determine the influence of FDs on side branches. All the lumbar arteries were covered and remained patent at 6- and 12-month angiography. Neointima hyperplasia was found along the wires of the FDs, and the ostia of the branches were partly covered. Wang et al. (21) placed an FD into the internal carotid artery of miniature pigs to assess the patency of collateral arteries, and reached a conclusion similar to that of Dai et al. (20).

Although our study indicates from hemodynamic analysis that posterior circulation branch arteries can be well-preserved after FD deployment, they may sometimes become occluded soon after therapy. Lall et al. (22) described 3 cases of branch occlusion immediately after pipeline embolization, one of which was a giant vertebrobasilar aneurysm involving the left PICA that was covered by a PED. Immediately after recovery from general anesthesia, the patient was found to have dysarthric speech as well as lower cranial neuropathy and had difficulty following commands. Emergency angiography was performed, and the left PICA was found to be occluded even though the patient was pre-medicated appropriately with anti-platelets, and genetic testing suggested clopidogrel responsiveness. After intra-arterial administration of abciximab, left PICA recanalization was observed on immediate angiography and at follow-up. To sum up, we speculate that it is not the mechanical blocking effect of the FD but thrombotic processes that contribute to acute occlusion. Furthermore, there are many reports of branch occlusion (23, 24), perforator infarctions (25), and ischemic strokes (1). More attention should thus be paid to the process of thrombogenesis.

There are several limitations to our study. First, limited specimen volume may reduce the reliability of this study. Second, patient-specific blood flow conditions were not available. Third, blood vessel walls were assumed to be rigid, which might not represent the true physiology of the human body. Fourth, most perforators are not visible in reconstruction geometry; we merely investigated the hemodynamic variation of AICAs and PICAs, which may have led to less than accurate results.

## Conclusion

Our findings have demonstrated that a flow diverter does not slow down the flow velocity of covered branches; in fact, flow velocity even increased in some cases. The coverage of posterior circulation branches of AICA, PICA, and other perforators is an acceptable approach if it is unavoidable. In addition, we found no linear relationship between branch diameter and flow reduction ratio. As this is a small case series, larger studies are needed to confirm these results.

## Data Availability

The datasets generated for this study are available on request to the corresponding author.

## Author Contributions

SM, XY, and YinZ contributed to conception and design of the study. XW, ZT, YaZ, and JC collected the data. XW and WL performed the statistical analysis. XW wrote the first draft of the manuscript. JL and YisZ wrote sections of the manuscript. All authors contributed to manuscript revision, and read and approved the submitted version.

### Conflict of Interest Statement

The authors declare that the research was conducted in the absence of any commercial or financial relationships that could be construed as a potential conflict of interest.

## References

[1] Brinjikji W Murad MH Lanzino G Cloft HJ Kallmes DF Endovascular treatment of intracranial aneurysms with flow diverters: a meta-analysis. Stroke. (2013) 44:442–7. 10.1161/STROKEAHA.112.678151 23321438

[2] Volker M Anastasios M Jan B Nuran A Thomas L Franziska D. Treatment of intracranial aneurysms with the pipeline embolization device only: a single center experience. Neurointervention. (2018) 13:32–40. 10.5469/neuroint.2018.13.1.32 29535896 PMC5847888

[3] Becske T Brinjikji W Potts MB Kallmes DF Shapiro M Moran CJ Long-term clinical and angiographic outcomes following pipeline embolization device treatment of complex internal carotid artery aneurysms: five-year results of the pipeline for uncoilable or failed aneurysms trial. Neurosurgery. (2017) 80:40–8. 10.1093/neuros/nyw014 28362885

[4] Becske T Kallmes DF Saatci I McDougall CG Szikora I Lanzino G. Pipeline for uncoilable or failed aneurysms: results from a multicenter clinical trial. Radiology. (2013) 267:858–68. 10.1148/radiol.13120099 23418004

[5] Zanaty M Chalouhi N Tjoumakaris SI Rosenwasser RH Gonzalez LF Jabbour P Flow-diversion panacea or poison? Front Neurol. (2014) 5:21 10.3389/fneur.2014.00021 24592254 PMC3938101

[6] Siddiqui AH Kan P Abla AA Hopkins LN Levy EI Complications after treatment with pipeline embolization for giant distal intracranial aneurysms with or without coil embolization. Neurosurgery. (2012) 71:E509–13; discussion E13. 10.1227/NEU.0b013e318258e1f8 22710418

[7] Fox B Humphries WE Doss VT Hoit D Elijovich L Arthur AS. Rupture of giant vertebrobasilar aneurysm following flow diversion: mechanical stretch as a potential mechanism for early aneurysm rupture. BMJ Case Rep. (2014) 2014:bcr2014011325. 10.1136/bcr-2014-011325 25355741 PMC4216898

[8] Cebral JR Mut F Weir J Putman C. Quantitative characterization of the hemodynamic environment in ruptured and unruptured brain aneurysms. AJNR Am J Neuroradiol. (2011) 32:145–51. 10.3174/ajnr.A2419 21127144 PMC3086563

[9] Paliwal N Yu H Xu J Xiang J Siddiqui A Yang X. Virtual stenting workflow with vessel-specific initialization and adaptive expansion for neurovascular stents and flow diverters. Comput Methods Biomech Biomed Engin. (2016) 19:1423–31. 10.1080/10255842.2016.1149573 26899135 PMC4945427

[10] Wang S Zhang Y Lu G Yang X Zhang X Ding G Hemodynamic performance of coil embolization and stentassisted coil embolization treatments: a numerical comparative study based on subject-specific models of cerebral aneurysms. Sci China Phys Mech Astronomy. (2011) 54:2053–63. 10.1007/s11433-011-4526-3

[11] Luo B Yang X Wang S Li H Chen J Yu H. High shear stress and flow velocity in partially occluded aneurysms prone to recanalization. Stroke. (2011) 42:745–53. 10.1161/STROKEAHA.110.593517 21233477

[12] Fan J Wang Y Liu J Jing L Wang C Li C. Morphological-hemodynamic characteristics of intracranial bifurcation mirror aneurysms. World Neurosurg. (2015) 84:114–20.e2. 10.1016/j.wneu.2015.02.038 25753233

[13] Stuhne GR Steinman DA. Finite-element modeling of the hemodynamics of stented aneurysms. J Biomech Eng. (2004) 126:382–7. 10.1115/1.1762900 15341176

[14] Malek AM Alper SL Izumo S. Hemodynamic shear stress and its role in atherosclerosis. JAMA. (1999) 282:2035–42. 10.1001/jama.282.21.2035 10591386

[15] Cebral JR Mut F Raschi M Scrivano E Ceratto R Lylyk P. Aneurysm rupture following treatment with flow-diverting stents: computational hemodynamics analysis of treatment. AJNR Am J Neuroradiol. (2011) 32:27–33. 10.3174/ajnr.A2398 21071533 PMC7964947

[16] Spetzler RF McDougall CG Zabramski JM Albuquerque FC Hills NK Russin JJ. The barrow ruptured aneurysm trial: 6-year results. J Neurosurg. (2015) 123:609–17. 10.3171/2014.9.JNS141749 26115467

[17] Lusseveld E Brilstra EH Nijssen PC van Rooij WJ Sluzewski M Tulleken CA. Endovascular coiling versus neurosurgical clipping in patients with a ruptured basilar tip aneurysm. J Neurol Neurosurg Psychiatr. (2002) 73:591–3. 10.1136/jnnp.73.5.591 12397159 PMC1738141

[18] Hu P Qian Y Zhang Y Zhang HQ Li Y Chong W. Blood flow reduction of covered small side branches after flow diverter treatment: a computational fluid hemodynamic quantitative analysis. J Biomech. (2015) 48:895–8. 10.1016/j.jbiomech.2015.02.015 25748223

[19] Mazur MD Kilburg C Wang V Taussky P. Pipeline embolization device for the treatment of vertebral artery aneurysms: the fate of covered branch vessels. J Neurointerv Surg. (2016) 8:1041–7. 10.1136/neurintsurg-2015-012040 26491041

[20] Dai D Ding YH Kadirvel R Rad AE Lewis DA Kallmes DF. Patency of branches after coverage with multiple telescoping flow-diverter devices: an *in vivo* study in rabbits. AJNR Am J Neuroradiol. (2012) 33:171–4. 10.3174/ajnr.A2879 22158925 PMC7966178

[21] Wang J Ding Y Wang Q Wang Y Mu S Bi L. The effect of placing flow-diverting stents in intracranial collateral arteries of miniature pig. Med Sci Monit. (2017) 23:1428–35. 10.12659/MSM.900622 28333907 PMC5374988

[22] Lall RR Crobeddu E Lanzino G Cloft HJ Kallmes DF. Acute branch occlusion after Pipeline embolization of intracranial aneurysms. J Clin Neurosci. (2014) 21:668–72. 10.1016/j.jocn.2013.07.011 24156905

[23] Szikora I Berentei Z Kulcsar Z Marosfoi M Vajda ZS Lee W. Treatment of intracranial aneurysms by functional reconstruction of the parent artery: the Budapest experience with the pipeline embolization device. AJNR Am J Neuroradiol. (2010) 31:1139–47. 10.3174/ajnr.A2023 20150304 PMC7963954

[24] Kulcsar Z Ernemann U Wetzel SG Bock A Goericke S Panagiotopoulos V. High-profile flow diverter (silk) implantation in the basilar artery: efficacy in the treatment of aneurysms and the role of the perforators. Stroke. (2010) 41:1690–6. 10.1161/STROKEAHA.110.580308 20616327

[25] Phillips TJ Wenderoth JD Phatouros CC Rice H Singh TP Devilliers L. Safety of the pipeline embolization device in treatment of posterior circulation aneurysms. AJNR Am J Neuroradiol. (2012) 33:1225–31. 10.3174/ajnr.A3166 22678845 PMC7965498

